# Determination of Ca, P, K, Na, and Mg in Australian Retail Pasteurised Milk Using Inductively Coupled Plasma Atomic Emission Spectroscopy (ICP OES)

**DOI:** 10.1155/2024/4417607

**Published:** 2024-08-12

**Authors:** Duc Doan Nguyen, Vicky Solah, Suzanne Daubney, Saijel Jani

**Affiliations:** ^1^ Food Science and Nutrition Centre for Crop and Food Innovation Food Futures Institute Murdoch University, Murdoch Western Australia 6150, Australia; ^2^ Food Science and Nutrition School of Medical, Molecular & Forensic Sciences College of Environmental & Life Sciences Murdoch University, Murdoch Western Australia 6150, Australia; ^3^ Bannister Downs Dairy, Northcliffe Western Australia 6262, Australia; ^4^ School of Maths, Statistics, Chemistry and Physics College of Science, Technology, Engineering, and Mathematics Murdoch University, Murdoch Western Australia 6150, Australia

## Abstract

A rapid and simple inductively coupled plasma atomic emission spectrometry (ICP OES) method was developed and validated for the determination of macroelements including calcium (Ca), phosphorus (P), potassium (K), sodium (Na), and magnesium (Mg) in Australian retail pasteurised milk. The milk samples were digested using the mixture of 70% HNO_3_ and 30% H_2_O_2_ (2 : 1, v/v) in an open-tube digester block at 120°C for 4 h. The validated ICP OES method showed good linearity for all elements (*R*^2^ > 0.9993). The method limits of quantification (LOQ) for Ca, P, K, Na, and Mg were 19.85, 8.97, 100.8, 41.92, and 11.56 *µ*g·g^−1^, respectively. Recoveries were in the range of 91.54–116.0%. Repeatability and interday reproducibility expressed as the relative standard deviation (% RSD) was below 5.0%. The contents of macroelements in 6 retail pasteurised milk samples were between 1099.32 and 1348.65 *µ*g·g^−1^ (Ca), 914.01 and 1091.21 *µ*g·g^−1^ (P), 1362.76 and 1549.74 *µ*g·g^−1^ (K), 288.89 and 323.22 *µ*g·g^−1^ (Na), and 97.62 and 110.57 *µ*g·g^−1^ (Mg). Principal component analysis (PCA) revealed that retail pasteurised milk samples were distinctly separated into four groups on the first two principal components (PCs). The difference in the macroelement content between milk brands might be affected by milk regions.

## 1. Introduction

Milk and milk products that are important sources of nutrients for human health contain 38 microelements and trace elements consisting of iron (Fe), zinc (Zn), selenium (Se), copper (Cu), manganese (Mn), iodine (I), molybdenum (Mo), and cobalt (Co) and macroelements such as calcium (Ca), phosphorus (P), potassium (K), sodium (Na), and magnesium (Mg) [1–4]. The mineral content in milk is influenced by the species, breed, individual animal, lactation stage, parity, udder health, feeding practices, and environmental conditions [2, 3, 5].

Milk and milk products are excellent sources of Ca, P, and Mg, providing approximately 34–44%, 23–32%, and 10–25% of daily intake in human diets, respectively [1]. Ca, P, and Mg are crucial elements found in the formation/maintenance of bones and teeth [2, 5, 6]. Ca is also associated with roles in muscle contraction, blood coagulation, and enzymatic reaction [1, 2, 4]. In addition, milk and milk products contribute 7% and 12% of daily Na and K intake to the human diet, respectively [7]. These elements play key roles in the cellular membrane potential and water balance in the body [8].

Drinking milk is a staple Australian food. Australians consume more than 2.4 billion litres of drinking milk, accounting for approximately 30% of raw milk between 2021 and 2022 [9]. In Australia, drinking milk is classified into ultrahigh temperature (long life), regular milk (full-fat milk), reduced fat (modified milk), and skim milk, which contain 3.8%, 3.5%, 1.2%, and 0.1% of fat (w/w), respectively [10]. The mineral content can also vary among drinking milk products. According to Food Standards Australia New Zealand, 100 mL of full-fat milk contains 120, 100, 166, 40, and 11 mg for Ca, P, K, Na, and Mg, respectively. Meanwhile, the same amount of skim milk (0.15% fat) contains 113, 85, 154, 36, and 10 mg for Ca, P, K, Na, and Mg, respectively [11, 12]. Recently, Dunshea et al. reported the content of Ca, P, K, Na, and Mg in 100 g of raw cow milk in Victoria, Australia, being 107.2, 88.5, 153.4, 34.7, and 9.8 mg, respectively [13]. To the best of our knowledge, however, there is little information about the content of macroelements in Australian retail pasteurised milk.

Sample preparation plays an important role in ICP OES analysis of elements in milk products. A method widely used for the digestion of milk samples is wet digestion involving strong acids and oxidized agents followed by heating at high temperatures. This digestion approach can be carried out using either an open-/closed-tube digester block/hot plate [14, 15] or a closed-tube microwave-assisted system [1, 3, 4, 13, 16, 17]. The closed-tube digester block/hot plate is a simple, cost-effective, and high-throughput option with a low risk of contamination. However, it is a time-consuming digestion approach. In contrast, microwave-assisted extraction (MAE) offers quicker digestion with a lower risk of contamination, but it requires expensive equipment equipped with a comprehensive safety system to avoid explosion [18]. Moreover, this digestion approach limits the number of samples that can be digested simultaneously and needs labour-intensive cleaning [19]. Alternatively, ultrasound-assisted extraction (UAE) can be applied for extracting elements in organic samples. It is a rapid, simple, and lower-cost sample preparation method to determine macroelements in coconut milk and rice-based infant formula [20, 21]. However, the effectiveness of UAE can be influenced by the physical characteristics of samples and the interactions between the analytes and the sample [20]. In milk, major amounts of macroelements such as Ca and P are present in colloidal calcium phosphate forms in casein micelles. This characteristic may be challenging when using UAE to determine Ca and P contents in milk samples using ICP OES.

An open-tube digestion system is advantageous for the elemental analysis of milk samples due to its simplicity, cost-effectiveness, and suitability for handling a large number of samples. However, this digestion technique has drawbacks, such as potential losses of elements or reagents due to volatilisation at high temperatures and a higher risk of contamination [18]. Tang et al. reported that using an open-tube digestion system with a mixture of HNO_3_ and H_2_O_2_ at 200°C for 4 h can lead to the loss or contamination of some elements when extracting elements from infant formula [15]. In contrast, Wheal et al. found the same efficiency between closed-tube digestion and open-tube digestion; both were used to extract elements from grain samples [19]. Similarly, Eliézer et al. reported similar elemental contents when milk powder samples were digested with a mixture of HNO_3_ and H_2_SO_4_ in an open-tube digester block at 120°C for 4 h and a microwave-assisted system [18]. However, using H_2_SO_4_ can have a strong negative impact on the environment. To the best of our knowledge, the extraction of macroelements from pasteurised milk samples with a mixture of HNO_3_ and H_2_O_2_ using an open-tube digester block at 120°C for 4 h has not yet been reported.

In this study, a rapid and simple ICP OES method was developed and validated to determine Ca, P, K, Na, and Mg content in retail pasteurised milk. An open-tube digester block was used to extract macroelements from milk samples. The proposed method was applied for the quantitative determination of these elements in 6 retail pasteurised milk made by different dairy processing plants around Australia.

## 2. Materials and Methods

### 2.1. Reagents and Standards

Analytical reagent-grade chemicals were used for all experiments. Nitric acid (HNO_3_, 70%) was purchased from Ajax Finechem (Vic, Australia). Hydrogen peroxide (H_2_O_2_, 30%) was purchased from ROWE Scientific (Wangara, WA, Australia). The stock standard solution of Ca (1000 mg·L^−1^) was a product of Scharlau (European Union). The stock solutions of P, K, Na, Mg, and Y (yttrium used as an internal standard; 10000 mg·L^−1^) were products of PerkinElmer (US). Working solutions were prepared by appropriately diluting the stock standard solutions in 2% impurity-free grade HNO_3_. All calibration solutions and the internal standard were prepared by adding 2% impurity-free grade HNO_3_ to working solutions to obtain the required concentrations.

### 2.2. Sample Collection

The infant formula (IF) with known contents of Ca, P, K, Na, and Mg from a reputable brand was collected from local supermarkets in Western Australia (WA) in March 2023. The IF sample was used immediately after opening. Six full-fat retail pasteurised milk (RPM) products were purchased from local supermarkets in May 2023. RPM samples were chosen to provide a snapshot of the milk available to Western Australian consumers. These milk samples were selected from the six largest dairy processing plants located in different regions in WA and New South Wales (NSW), Australia.

After being transported to the laboratory, RPM samples were immediately digested with the mixture of 70% HNO_3_ and 30% H_2_O_2_ (Section 2.3). The chemical composition of RPM and IF as reported by the manufacturers is presented in Table 1.

### 2.3. Sample Digestion

#### 2.3.1. Infant Formula Digestion

The digestion was carried out as described by Eliézer et al. [18] with some modifications. Before preparing samples, all glassware was soaked in 10% HNO_3_ overnight, and rinsed with Milli-Q water, followed by air dried at room temperature until dryness. 0.1 gram of the IF sample weighed accurately (±0.0001 g) was carefully placed in the bottom of the Pyrex tube. After gradually adding 5 mL of 70% HNO_3_ and 2.5 mL of 30% H_2_O_2_ to the edge of the Pyrex tube, the mixture was gently and thoroughly mixed.

All Pyrex tubes were uncapped and placed in the digester block, which was placed in a fume cabinet. The digestion was performed at 120°C for 4 h. An external thermometer was used to ensure that the temperature in the digester block was maintained at 120°C throughout the digestion process. The mixture of 5 mL of 70% HNO_3_ and 2.5 mL of 30% H_2_O_2_ treated in the same manner but without IF was used as a reagent blank. The digested samples and the reagent blank were cooled down to room temperature, then diluted to 25 mL with Milli-Q water, and filtered through Whatman paper N^o^1 before ICP OES analysis. The IF sample was digested in four replicates.

#### 2.3.2. Retail Pasteurised Milk Digestion

1 mL of each RPM sample carefully placed at the bottom of the Pyrex tube was weighed accurately (±0.0001 g). Following this, the digestion process was performed as described in Section 2.3.1. The reagent blanks were treated in the same digestion procedure for RPM samples but without the milk. RPM samples were digested in triplicates.

### 2.4. Method Validation

The method validation was carried out as described by Pekou et al. [14]. The ICP OES method was validated in terms of linearity, method limits of detection (LOD), method limits of quantification (LOQ), recovery, and precision (repeatability and interday reproducibility).

For method linearity evaluation, calibration curves for Ca, P, K, Na, and Mg were constructed by analysing standard solutions at different concentrations. LOD and LOQ were estimated by consecutively injecting the reagent blanks at 6 repetitions into the ICP OES system. LOD and LOQ were calculated based on 3 and 10 times the standard deviation of the reagent blanks divided by the slope of the calibration curves, respectively. LOD and LOQ were converted in *µ*g·g^−1^ based on the sample weight of IF (0.1 gr) and the volume of the diluted sample (25 mL).

Recovery expressed as percent (%) was assessed by comparing the contents of Ca, P, K, Na, and Mg in the IF sample measured by the ICP OES method to the values reported by the manufacturer. Repeatability was evaluated by measurement of repeated four digested IF samples, while interday reproducibility was evaluated by measurement of repeated four digested IF samples on two different days. Both were expressed as relative standard deviations (%RSDs).

### 2.5. ICP OES Analysis

Ca, P, K, Na, and Mg in digested milk samples and standard solutions were determined using ICP OES iCAP 7600 series (Thermo Scientific, USA). The instrumental conditions are summarised in Table 2.

Regarding the spectral lines, the analytical wavelengths monitored for each element are presented in Table 3. The amount of Ca, P, K, Na, and Mg were quantified based on standard calibration curves and intensities of each element.

### 2.6. Data Analysis

Data for macroelements of RPM samples were expressed as the mean ± standard deviation (mean ± SD). Principal component analysis (PCA) was applied for the visualisation of the discrimination in the content of macroelements between milk brands. Six RPM samples (6 milk brands) and 5 elements (Ca, P, K, Na, and Mg were variables) were taken into consideration. The data matrix was constructed with rows representing 6 milk brands and columns representing 5 variables. Data were analysed using R software (version 4.2.2) installed with two packages of FactoMineR and factoextra.

## 3. Results and Discussion

### 3.1. ICP OES Performance

Ca, P, K, Na, and Mg in digested milk samples and standard solutions were determined using the ICP OES iCAP7600 series (Thermo Scientific, USA). Radio frequency (RF) power plays a vital role in operating ICP OES plasma [4]. RF power and other performance conditions previously routinely optimised are shown in Table 2. To select sensitive spectral lines, standard solutions at different concentrations of Ca, P, K, Na, and Mg were analysed at different wavelengths and view types (radial or axial). Because cow milk contains various elements, some of which can interfere with target analytes, therefore, a digested pasteurised milk sample was also analysed for Ca, P, K, Na, and Mg to select sensitive spectral lines. In the present study, optimal spectral lines and view type for each element were selected based on their intensities and the absence of interference (Table 3).

### 3.2. Method Validation

Ideally, certified reference materials for liquid milk/infant formula would have been used to evaluate the accuracy of the proposed method. However, these materials were very high cost or limited in Australia due to quarantine import restrictions. It is acknowledged that the chemical composition of IF, including the mineral content, requires rigorous regulatory control [22], particularly elements in IF intended for feeding infants aged 0–6 months. The mineral content in IF produced by reputable manufacturers can be highly reliable. Therefore, we used the IF sample from a reputable brand to validate the ICP OES method in the present study.

The results showed that the ICP OES method had good linearities with *R*^2^ ranging from 0.9993 to 1.000 for all elements. The recovery ranged from 91.54% to 116.00%. LOD ranged from 2.63 to 30.20 *µ*g·g^−1^, while LOQ was between 8.97 and 100.80 *µ*g·g^−1^ (Table 4).

The results also showed that repeatability was from 0.68 to 3.50%, while interday reproducibility ranged from 3.12 to 5.00% (Table 5). Therefore, this validated method was considered satisfactory and applied for the quantitative determination of Ca, P, K, Na, and Mg contents in RPM.

### 3.3. Macroelements Composition in Retail Pasteurised Milk

The proposed ICP OES method was used to simultaneously determine Ca, P, K, Na, and Mg content in 6 RPM samples. Ca, P, K, Na, and Mg contents are presented in Table 6.

The most abundant macroelement found in all milk samples was K (1362.76–1549.74 *µ*g·g^−1^), followed by Ca (1099.32–1348.09 *µ*g·g^−1^) and P (914.01–1091.21 *µ*g·g^−1^). The highest content for K was quantified in RPM6 (1549.74 *µ*g·g^−1^), while the lowest content was obtained from RPM3 (1362.76 *µ*g·g^−1^). Similarly, the highest contents of Ca and P were observed in RPM6 (1348.56 and 1091.21 *µ*g·g^−1^, respectively), whereas the lowest contents were found in RPM3 (1099.32 and 914.01 *µ*g·g^−1^, respectively). As shown in Table 6, the average contents of K, Ca, and P observed in Australian pasteurised milk in the present study were 1455.42, 1174.84, and 991.93 *µ*g·g^−1^, respectively. These results are higher than values previously reported in the literature [1, 4, 23]. However, the content of Ca is below the value reported by Lopez et al. [24].

The highest content of Na was found in RPM6 (323.22 *µ*g·g^−1^) followed by in RPM4 (318.31 *µ*g·g^−1^) and in RPM5 (302.33 *µ*g·g^−1^). The lowest content was observed in RPM2 (288.89 *µ*g·g^−1^). In the present study, Mg was the least predominant macroelement found in all milk samples with its content ranging from 97.62 *µ*g·g^−1^ in RPM2 to 110.57 *µ*g·g^−1^ in RPM6. As shown in Table 6, the average contents of Na and Mg in Australian pasteurised milk observed in the present study were 304.03 and 101.39 *µ*g·g^−1^, respectively. These results are similar to values reported by Newton et al. [1] but are lower than those reported by Ahmed et al. [4].

To the best of our knowledge, there has been a limited study reporting the content of K, Ca, P, Na, and Mg in pasteurised milk in Australia. In recent years, Dunshea et al. investigated minerals in cow milk collected from dairy farms in Victoria, Australia [13]. Compared to the results of the present study, the average content of K (1534 *µ*g·g^−1^) and Na (347 *µ*g·g^−1^) are markedly higher, whereas Ca (1072 *µ*g·g^−1^), P (885 *µ*g·g^−1^), and Mg (98 *µ*g·g^−1^) are slightly lower.

**Figure 1:**
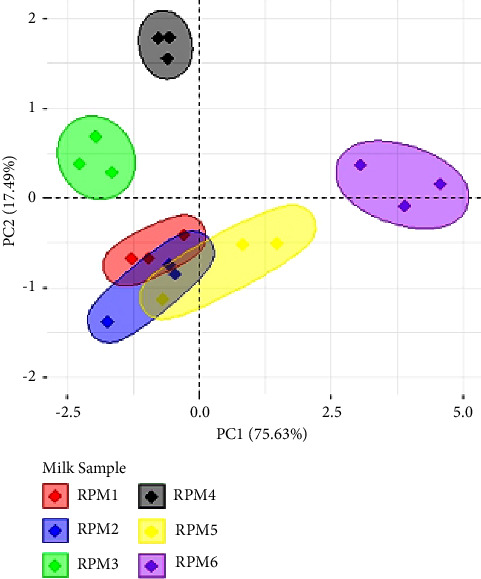
Principal component analysis for macroelements composition in milk.

PCA analysis revealed that the RPM samples distinctly separated into four groups on the first two principal components (PCs). The first two PCs explained 93.12% of the total variance in the original dataset (PC1 explained 75.63% of the total variance and PC2 explained 17.49% of the total variance) (Figure 1). Notably, RPM1, RPM2, and RPM5 exhibited slight overlap and formed a distinct group, while RPM3, RPM4, and RPM6 each separated into three other groups (Figure 1).

To sum up, thermal treatment plays a crucial role in milk processing, impacting both shelf-life and nutrients. The pasteurisation process can lead to some nutrient losses but does not significantly alter Ca, P, K, Na, and Mg content in milk [2]. It is acknowledged that milk mineral content depends on species, breed, individual animal, lactation stage, parity, and udder health [3, 5]. In addition, the differences in macroelement contents can be attributed to elemental composition of soil, water, and plants in dairy farms, which in turn affect the feed composition [17].

Notably, all RPM samples in the present study were collected from local markets in late Autumn in Australia, specifically in May 2023. It was assumed that cows were grazing during this period. Consequently, the observed variation in macroelement contents between milk brands may be attributed to differences in regions where dairy farmers, whose milk was supplied to processing plants, were located.

## 4. Conclusions

A simple and rapid ICP OES method was successfully developed and validated to quantitatively determine Ca, P, K, Na, and Mg contents in RPM. An open-tube digester block used to digest milk samples in a mixture of HNO_3_ and H_2_O_2_ at 120°C for 4 h was an efficient digestion approach for the extraction of macroelements. This study provides a simple, cost-effective, and high-throughput digestion method for the simultaneous determination of macroelements in pasteurised milk by ICP OES. However, it is a time-consuming digestion approach. Results showed that K (1455.42 *µ*g·g^−1^) was the most abundant element, while Mg (101.39 *µ*g·g^−1^) had the lowest content in Australian pasteurised milk. The content of 5 macroelements varied depending on the milk brands, which may be attributed to differences in milk regions. The results obtained in the present study are valuable information about macroelements to dairy consumers, farmers, and processors. However, the number of RPM samples investigated in the present study was mainly collected in WA, Australia. Therefore, further studies are needed to elaborate on a large number of milk samples across Australia to obtain comprehensive information about the mineral content.

## Figures and Tables

**Table 1:** The chemical composition of retail pasteurised milk and infant formula (g 100 mL^−1^).

Retail pasteurised milk	Protein	Fat	Carbohydrate	Elements				
				Ca	P	K	Na	Mg
RPM1	3.4	3.4	4.4	118.0	84	143	44	—
RPM2	3.4	1.2	5.0	128.0	—	—	41	—
RPM3	3.3	3.6	4.9	120.0	—	—	40	—
RPM4	3.8	4.0	4.9	129.0	—	—	37	—
RPM5	3.3	3.5	5.0	109.0	—	147	33	—
RPM6	3.8	4.8	4.6	135.8		153	38	
IF^*∗*^	1.5	3.1	8.3	53.0	32	78	30	6.8

^*∗*^According to a manufacturer, 136 g of infant formula powder (IF) and 900 mL of water make 1000 mL of liquid formula.

**Table 2:** Instrumental conditions for ICP OES.

Parameters	Unit	Value
RF power	W	1150
Auxiliary gas flow	L·min^−1^	0.5
Nebulizer gas flow	L·min^−1^	0.7
Coolant gas flow	L·min^−1^	12
Nebulizer pressure	kPa	450
Peristaltic pump	Rpm	45

**Table 3:** The spectral lines for the analysis of macroelements.

Elements	Wavelength (nm)	View type
Ca	396.847	Radial
P	177.495	Axial
K	769.896	Radial
Na	589.592	Radial
Mg	279.553	Axial

**Table 4:** Linearity, limit of detection, and limit of quantification.

Elements	Standard solution ranges (*µ*g·mL^−1^)	Calibration curves	*R*-square	LOD (*µ*g·g^−1^)	LOQ (*µ*g·g^−1^)
Ca	5–50	*y* = 35259*x* + 30791	0.9993	5.95	19.85
P	5–50	*y* = 143.91*x* + 59.82	0.9998	2.63	8.97
K	5–100	*y* = 105.71*x* + 60.25	0.9999	30.20	100.8
Na	0.1–25	*y* = 875.09*x* + 210.87	1.0000	13.22	41.92
Mg	0.1–10	*y* = 136988*x* + 18672	0.9996	3.47	11.56

LOD: limit of detection, LOQ: limit of quantification.

**Table 5:** Recovery, repeatability, and interday reproducibility.

Elements	Values reported by a manufacturer (mg·g^−1^)	Measured values (mg·g^−1^)	Recoveries (%)	Repeatability (% RSD)	Interday reproducibility (% RSD)
Ca	3.90	3.57 ± 0.10	91.54	2.69	3.12
P	2.35	2.35 ± 0.02	100.00	0.68	4.32
K	5.74	5.50 ± 0.05	95.82	0.97	4.31
Na	2.21	2.20 ± 0.04	99.55	1.60	3.46
Mg	0.50	0.58 ± 0.02	116.00	2.65	4.50

RSD: relative standard deviation.

**Table 6:** The content of macroelements in retail pasteurized milk.

Retail pasteurised milk	The content of elements^a^ (*µ*g·g^−1^)				
	Ca	P	K	Na	Mg
RPM1	1134.00 ± 14.78	978.96 ± 7.88	1449.73 ± 15.96	292.23 ± 3.16	99.51 ± 2.18
RPM2	1146.46 ± 22.47	982.67 ± 4.79	1461.85 ± 31.61	288.89 ± 9.00	97.62 ± 1.00
RPM3	1099.32 ± 17.06	943.00 ± 9.99	1362.76 ± 22.37	299.22 ± 2.70	97.76 ± 1.71
RPM4	1119.34 ± 23.59	914.01 ± 13.83	1421.98 ± 13.41	318.31 ± 3.08	102.34 ± 1.54
RPM5	1183.84 ± 31.97	1041.75 ± 15.68	1486.46 ± 39.81	302.33 ± 9.46	100.51 ± 2.91
RPM6	1348.09 ± 13.65	1091.21 ± 45.08	1549.74 ± 2.63	323.22 ± 2.11	110.57 ± 4.46
Average	1171.84	991.93	1455.42	304.03	101.39

^a^Mean ± standard deviation (*n* = 3).

## Data Availability

The data used to support the findings of this study are included within the article.
